# Novel Silicone-Coated ^125^I Seeds for the Treatment of Extrahepatic Cholangiocarcinoma

**DOI:** 10.1371/journal.pone.0147701

**Published:** 2016-02-03

**Authors:** Lizhou Lin, Lili Guo, Weixing Zhang, Xiaobo Cai, Dafan Chen, Xinjian Wan

**Affiliations:** Department of Gastroenterology, Shanghai First People’s Hospital, School of Medicine, Shanghai Jiaotong University, Shanghai, 200080, People’s Republic of China; Brandeis University, UNITED STATES

## Abstract

^125^I seeds coated with titanium are considered a safe and effective interstitial brachytherapy for tumors, while the cost of ^125^I seeds is a major problem for the patients implanting lots of seeds. The aim of this paper was to develop a novel silicone coating for ^125^I seeds with a lower cost. In order to show the radionuclide utilization ratio, the silicone was coated onto the seeds using the electro-spinning method and the radioactivity was evaluated, then the anti-tumor efficacy of silicone ^125^I seeds was compared with titanium ^125^I seeds. The seeds were divided into four groups: A (control), B (pure silicone), C (silicone ^125^I), D (titanium ^125^I) at 2 Gy or 4 Gy. Their anti-tumour activity and mechanism were assessed in vitro and in vivo using a human extrahepatic cholangiocarcinoma cell line FRH-0201 and tumor-bearing BALB/c nude mice. The silicone ^125^I seeds showed higher radioactivity; the rate of cell apoptosis in vitro and the histopathology in vivo demonstrated that the silicone ^125^I seeds shared similar anti-tumor efficacy with the titanium ^125^I seeds for the treatment of extrahepatic cholangiocarcinoma, while they have a much lower cost.

## Introduction

Worldwide, cholangicarcinoma(CC) is the second commonest primary liver cancer after hepatocellular carcinoma, and accounts for 15% of all primary hepatic malignancies[1–2]. With the incidence and mortality rates risen in extrahepatic CC (including perihilar cholangicarcinoma), the diagnosis rates for CC have risen steeply and steadily across the world over the past few decades. Currently, the only curative therapeutic option in extrahepatic CC is resection. However, only a minority of the patients can be operated and even if a clear resection (R0 resection) is possible, the rate of relapse is as high as 60–75% [2–5]. Although multimodal therapeutic concepts have been proposed, they were not successful to achieve major progress.Hence, the insertion of biliary stents has been widely accepted as a mainly palliative procedure for the improvement of biliary drainage[5–9]; however, the prognosis remains poor, with complex hilar lesions conferring a median patient survival of less than 6 months[10–11]. Since the cause of death in extrahepatic CC is commonly due to recurrent biliary obstruction and intrabiliary sepsis, key issues are controlling local disease and optimizing biliary drainage[1]. There is evidence indicating that the combination of radiotherapy with stent insertion improves survival time compared to stent insertion alone[12–13].

For decades, ^125^I seeds have been successfully used as interstitial brachytherapy for prostate, pancreatic, and lung cancer, where they are characterized by a long half-life of 59.6 days[14–16]. Liu et al. first introduced endoprosthesis containing ^125^I seeds for intraluminal brachytherapy in the biliary system, and demonstrated that the combination of ^125^I seeds and stenting was a useful and well-tolerated method for treatment of advanced extrahepatic CC[17].The conventional ^125^I seeds are coated by titanium, it is a mental material which has the characteristics of no toxicity, undegradable and good biocompatibility. However, the titanium coating ^125^I seeds are quite expensive, and approximately twice the amount of ^125^I solution is required to obtain effective radioactivity due to the self-shielding effect of the titanium coating, further increasing the price. Therefore, developing simple and cheap coatings for ^125^I seeds may hold therapeutic promise. Medical silicone is widely used for biomedical purposes, such as coating neural microelectrodes and antibiotics. The medical silicone has many advantages, beside the capability of corrosion resistance, the suitable biocompatibility and appropriate absorption rate make it becomes widely used medical material[18–20]. However, there have been few studies into the use of ^125^I-coated-silicone seeds for tumor radiotherapy.

The aim of this study was to develop silicone coating for ^125^I seeds and compare the safety and efficacy with conventional titanium coated ^125^I seeds for the treatment of extrahepatic CC.

## Materials and Methods

### 2.1. Coating technique

**Fig 1:**
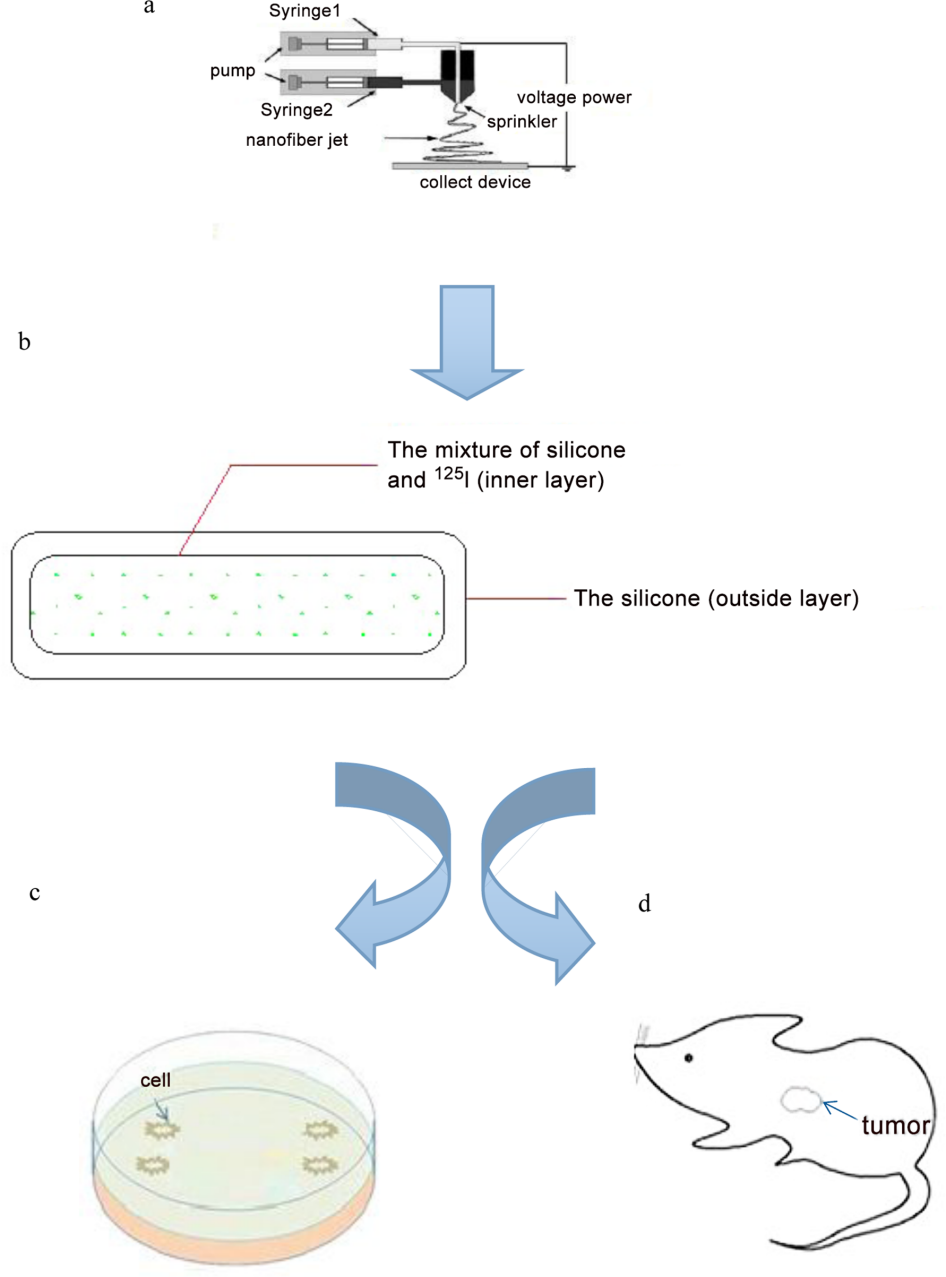
Diagrams illustrating the experimental design: (a) coaxial electrospinning device, silver-silicone with ^125^I in syringe1, another silicone in syringe2 as the shell, the jets forming the core—shell structure; (b) the principle diagram of seeds, the outside layer is the silicone coating the ^125^I solution inside(c,d) the anti-tumor effect of the silicone was examined in vitro and in vivo using human cell line FRH-0201 and tumor-bearing BALB/c nude mice.

**Fig 2:**
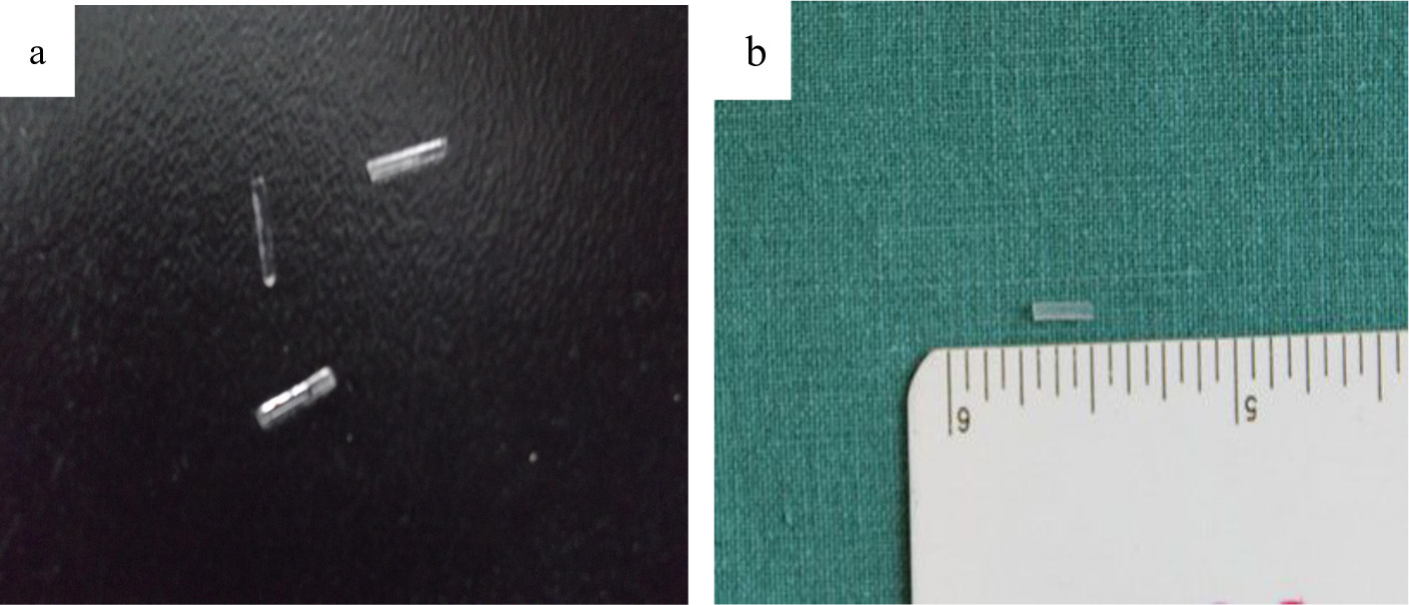
Silicone ^125^I seeds, the diameter and length was 0.8 mm by 4.5 mm.

Silicone with a pore size of 10 A was dissolved in silver nitrate solution to form silver-silicone, and the solution was subject to spinning. The ^125^I solution (specific activity greater than 800 mCi/ml) was added into the spinning solution, which was immediately discharged into a syringe. Another syringe was filled with the silicone solution. The syringes were connected to a high voltage power supply controlled at 25 kV in a modified coaxial electrospinning device (Cole-Parmer Instrument Company, USA) as shown in Fig 1. The solution was sprayed evenly onto a nanofiber mixture to form an initial silicone ^125^I (s-^125^I) mixture. The nanofibers were collected on aluminum foil, forming a core-shell structure, with the core composed of silver-silicone containing ^125^I and a shell of silicone. Nanofibers were then cut into seeds. All processes were undertaken in a sealed and protective glove box. All samples were vacuum dried at 60°C for 12 h to remove solvents. Following solvent removal, the seeds received an appearance inspection, and were cleaned in scintillation fluid (Fig 2). The cleaning fluid was collected to check for leaks (less than 185 Bq is qualified). Finally, the radioactivity of each seeds was measured, with recordings of radioactivity taken for 20 groups (Table 1). The utilization of the radionuclide was estimated using Eq 1.

**Table 1:** 20 groups datas of silicone ^125^I seeds and titanium ^125^I seeds.

Encapuslate material	Titanium			Silicone		
Total radioactivity of ^125^I solution		24mCi^*^			24mCi	
		Radioactivity^Δ^			Radioactivity	
	NO.	μCi	KBq	NO.	μCi	KBq
	1	575	21275	1	1092	40404
	2	468	23458	2	1047	38739
	3	558	20646	3	1250	46250
	4	778	20498	4	1211	44807
	5	625	23125	5	1158	42846
	6	472	24272	6	1202	44474
	7	548	20276	7	1057	39109
	8	635	23495	8	1125	41625
	9	722	20017	9	1215	44955
	10	528	25641	10	908	33596
	11	713	26381	11	897	33189
	12	541	26714	12	1209	44733
	13	554	28786	13	1308	48396
	14	717	26529	14	928	34336
	15	523	19351	15	1306	48322
	16	693	19536	16	879	32523
	17	595	22015	17	913	33781
	18	496	18352	18	1264	46768
	19	656	17464	19	952	35224
	20	634	17316	20	957	35409
Total		12031	445147		21878	809486

*1mCi = 182Gy = 37MBq

Δ indicated the final radioactivity corrected to the same day

R(%)=A/T (1)

Where R means radionuclide utilization ratio; A is the actual ^125^I radioactivity; T is the total ^125^I radioactivity.

The titanium ^125^I(t-^125^I) seeds were produced by XinKe Co., China. The diameter and length of each titanium capsule was 0.8 mm by 4.5 mm.

Compare the radionuclide utilization ratio and radioactivity of s-^125^I with t-^125^I.

### 2.2 Cell apoptosis evaluation in vitro

**Fig 3:**
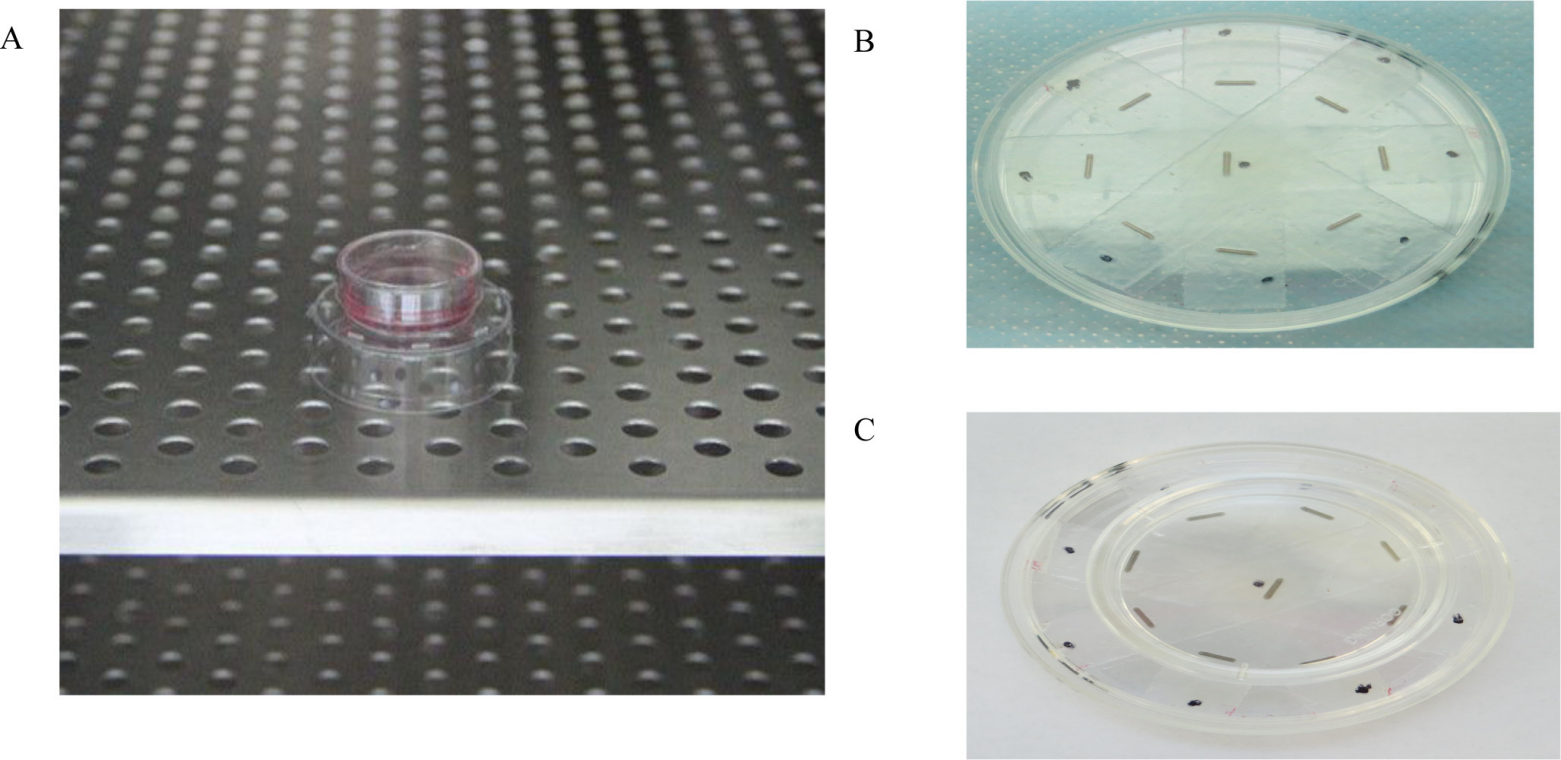
A.The irradiation model was placed in the incubator. B. Eight ^125^I seeds were fixed on the dish around the 35 mm diameter circumference, with one ^125^I seed placed at the center of dish. C. A 35mm culture dish was placed on the top of the irradiation model.

To obtain a relatively homogeneous dose distribution at the top of the dish, eight ^125^I seeds were evenly fixed with double-sided adhesive around a 35 mm diameter circle in the center of a 6-cm cell culture dish, with one ^125^I seed placed in the center (Fig 3B). A 35-mm cell culture dish containing cells was placed on the top of the ^125^I seed irradiation model during the experiment (Fig 3C). The model was kept in the incubator to maintain constant cell culture conditions (Fig 3A) and was validated with thermoluminescent dosimetry measurement using an empirical formula established by the American Association of Physicists in Medicine (AAPM; 15)[21]. The exposure time for delivering doses of 2 Gy and 4 Gy were 44 h and 92 h, respectively.

The human extrahepatic cholangiocarcinoma cell line FRH-0201 was provided by the Central Laboratory of First People’s Hospital (Shanghai, China), were maintained in Roswell Park Memorial Institute (RPMI) 1640 medium (Gibco, Invitrogen) supplemented with 10% fetal bovine serum (Gibco, Invitrogen) and 1% pen-strep (100 U/ml penicillin and 100 mg/ml streptomycin) (Gibco, Invitrogen) in a 37°C humidified incubator containing 5% CO_2_. When the cell concentration reached 1 × 10^5^ cells/mL, cells were placed at the top of the ^125^I seed irradiation model in the incubator. The cells were randomly divided into four groups: group A (control), B (pure silicone, only silicone), C (t-^125^I), and D (s-^125^I), and maintained in culture above the seeds for lengths of time equivalent to 2 and 4 Gy doses.

Apoptosis analysis using flow cytometry: Following exposure to radioactive seeds, 5 x 10^5^ cells were collected from each group and washed twice with phosphate buffered saline. The cell pellet was resuspended in 500 mL ice-cold binding buffer. Then, 5 μL of Annexin V-FITC and 100 uL of propidium iodide solution (Becton-Dickinson, BD, USA) were added to the cell suspension, and mixed by mild vortexing. The samples were incubated for 15 min in the dark before flow cytometric analysis. Stained cells were analyzed with fluorescence-activated cell sorting (BD Biosciences, USA). The assay was performed 3 times for each experimental group.

### 2.3 Animal preparation

A total of 24 female athymic BALB/c nude mice (6–8 weeks old) weighing 18–20g were supplied by the Laboratory Animal Center of Shanghai First People’s Hospital (Shanghai, China). These mice received humane care in a pathogen-free environment (23 ± 2°C and 55% ± 5% humidity). They were maintained on a 12-h light and 12-h dark cycle, with food and water supplied during the entire experimental period. Animal care and surgery protocols were approved by the Animal Care Committees of Shanghai Jiaotong University.

In order to establish xenograft tumors, 2 × 10^5^ FRH-0201 cells were subcutaneously injected into the back of the BALB/c nude mice. After 16 days, all mice had tumors with a volume of approximately 100 mm^3^, then were randomly separated into four groups: A (control), B (pure silicone), C (t-^125^I), and D (s-^125^I), three per group according to the volume of tumor calculated by Eq 2 [21]. Tumor volume and mouse weight change ratio (%) were calculated using Eqs 3 and 4, respectively.

$$V=L\times W^{2}\;/\;2$$ (2)

$$\text{Tumor volume change ratio}\left(\%\right)=\;(V_{1}/V_{N}-1)\times 100\%$$ (3)

$$\text{Mouse weight change ratio}\;\left(\%\right)=\;(W_{1}/W_{N}-1)\times 100\%$$ (4)

where V is the tumor volume; L is the tumor length; W is the tumor width; V_1_ is the tumor volume measured on the first day; and V_N_ is the tumor volume measured N days after implantation with ^125^I seeds; W_1_ is the mouse weight measured on the first day; and W_N_ is the mouse weight measured N days after treatment with ^125^I seeds.

**Fig 4:**
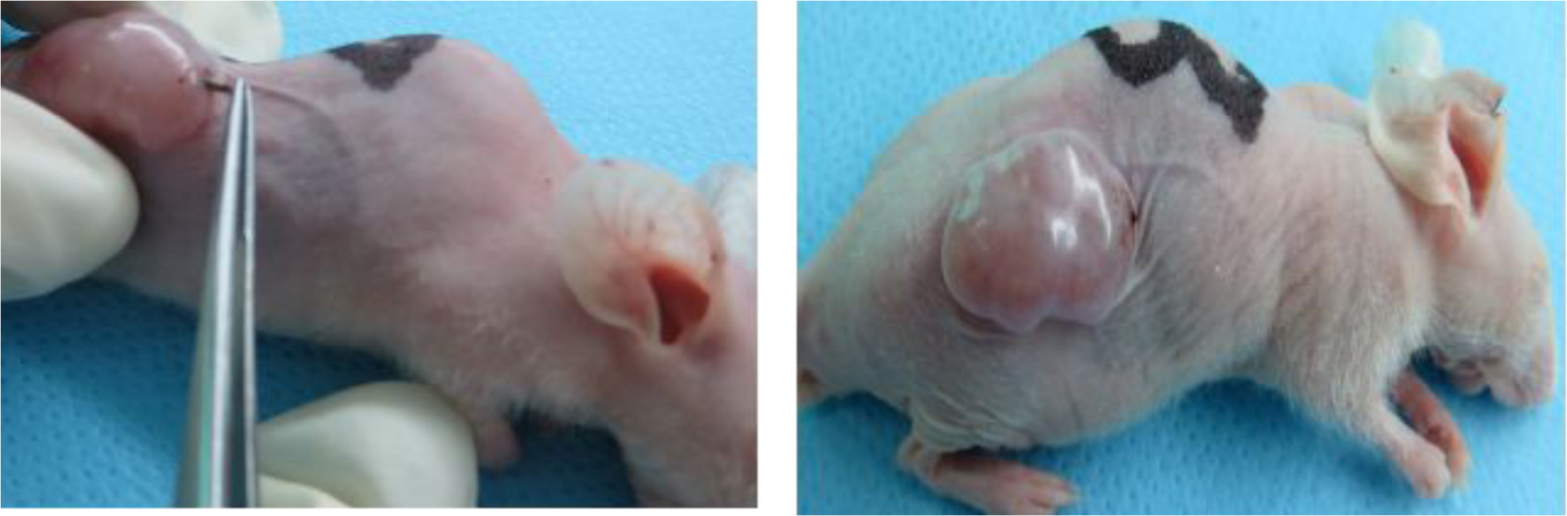
Operative procedure for the seed implantation into the tumor: (a) the mouse was given a implantation of seed by tweezers; (b) the seed was implanted into the tumor.

All the mice were administered general anesthesia by intraperitoneal injection of pentobarbital sodium, and an 18-gauge needle was used to implant ^125^I seeds, a straight

skin incision, approximately 1 cm long, into the tumour was performed so that sufficient space was created for embedding the ^125^I seed. Finally, the incision was sutured so that the tumor could be treated locally.Fig 4 shows the operative procedure for the seed implantation into the tumor. All tumors volume and mouse weight were measured every two days, and the mice were humanely sacrificed after 16 days.

### 2.4 TUNEL staining

The fresh tumor tissues were fixed in 10% formalin solution and embedded in paraffin. Formalin-fixed, paraffin embedded blocks were sectioned at 5 mm, and the sections were baked for 1 h at 60°C. The tissue sections were incubated in 0.1% Triton X-100 in 0.1% sodium citrate for 15 min, followed by incubation in 0.3% H_2_O_2_ for 30 min. The slides were washed three times in phosphate-buffered saline and incubated with 50 μL of TUNEL reaction mixture (TdT and fluorescein-labeled dUTP) in a dark and humid atmosphere for 60 min at 37°C. Slides were mounted in neutral gum medium, and 100 fields were observed with a fluorescence microscope (Olympus, Tokyo, Japan). Pictures were taken with an MZ FLIII stereomicroscope (Leica Microsystems, Deerfield, IL) with red-field transmitted light, and apoptotic cells were counted.

### 2.5 Hematoxylin and eosin staining

All tumors were fixed in 10% formalin solution, and embedded in paraffin. Formalin-fixed, paraffin-embedded blocks were sectioned at 5 mm, and hematoxylin and eosin stained for examination. A slide of each tumor was evaluated and imaged at a high magnification (×50 and ×200). Representative images were captured with a Leica microscope (Leica Microsystems, Wetzlar, Germany).

### 2.6 Statistical analyses

All statistical analyses were performed with SPSS software, version 13.0 for Windows (SAS Institute, Cary, NC). Data are presented as mean ± standard deviation. Student’s t-tests were used to compare apoptosis of tumor cells, as well as the volume and weight change before and after treatment. Student’s t-test was also used to compare the number of apoptotic cells following TUNEL staining. Because multiple comparisons were performed on some data, it was noted wherever statistical significance would be removed by using the Bonferroni method. Further, p < 0.05 was considered statistically significant.

## Result

### 3.1 Utilization of ^125^I radionuclides

**Fig 5:**
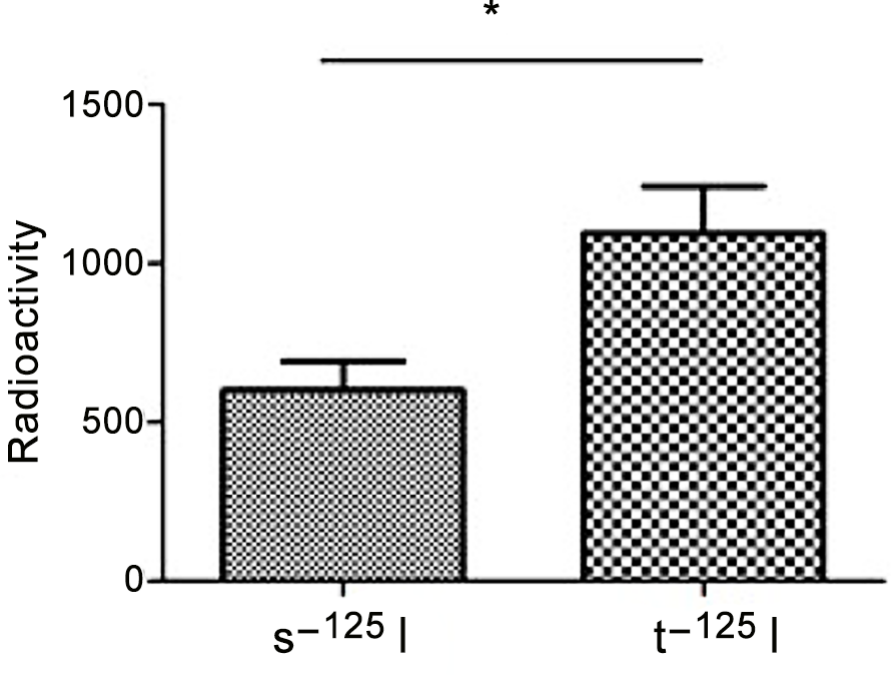
The radioactivity comparison of s-^125^I seeds and t-^125^I seeds. The radioactivity of s-^125^I is higher than t-^125^I obviously.

With the help of a coaxial electrospinning device, the production of silicone ^125^I seed was straightforward, the radioactivity of s-^125^I seeds were obviously higher than the t-^125^I seeds (Fig 5), and the radionuclide was well dispersed within the silicon-silver. As shown in Table 2, ^125^I is highly utilized by the silicone seeds. According to Eq 1, the utilization of ^125^I by silicone was highly improved, with 91.2% of the ^125^I being utilized, while the titanium only utilized 50.1% of the ^125^I. Throughout all procedures, safe handling of the radionuclide was prioritized, and the remaining solution required less than 185 Bq (5 nCi).

**Table 2:** The radioactivity and radionuclide utilization ratio comparison of s-^125^I Seeds and t-^125^I Seeds.

Sample	t-^125^I seeds	t-^125^I seeds	*P* value
	(n = 20)	(n = 20)	
**Radioactivity**	**601.55±90.09**	**1093.90±149.51**	<0.05*
**Radionuclide utilization ratio (%)**	**50.1%±7.40(%)**	**91.2%±6.47(%)**	

*p<0.*05* means there was statistically significant difference between the two coating seeds.

### 3.2 Apoptosis analysis

**Fig 6:**
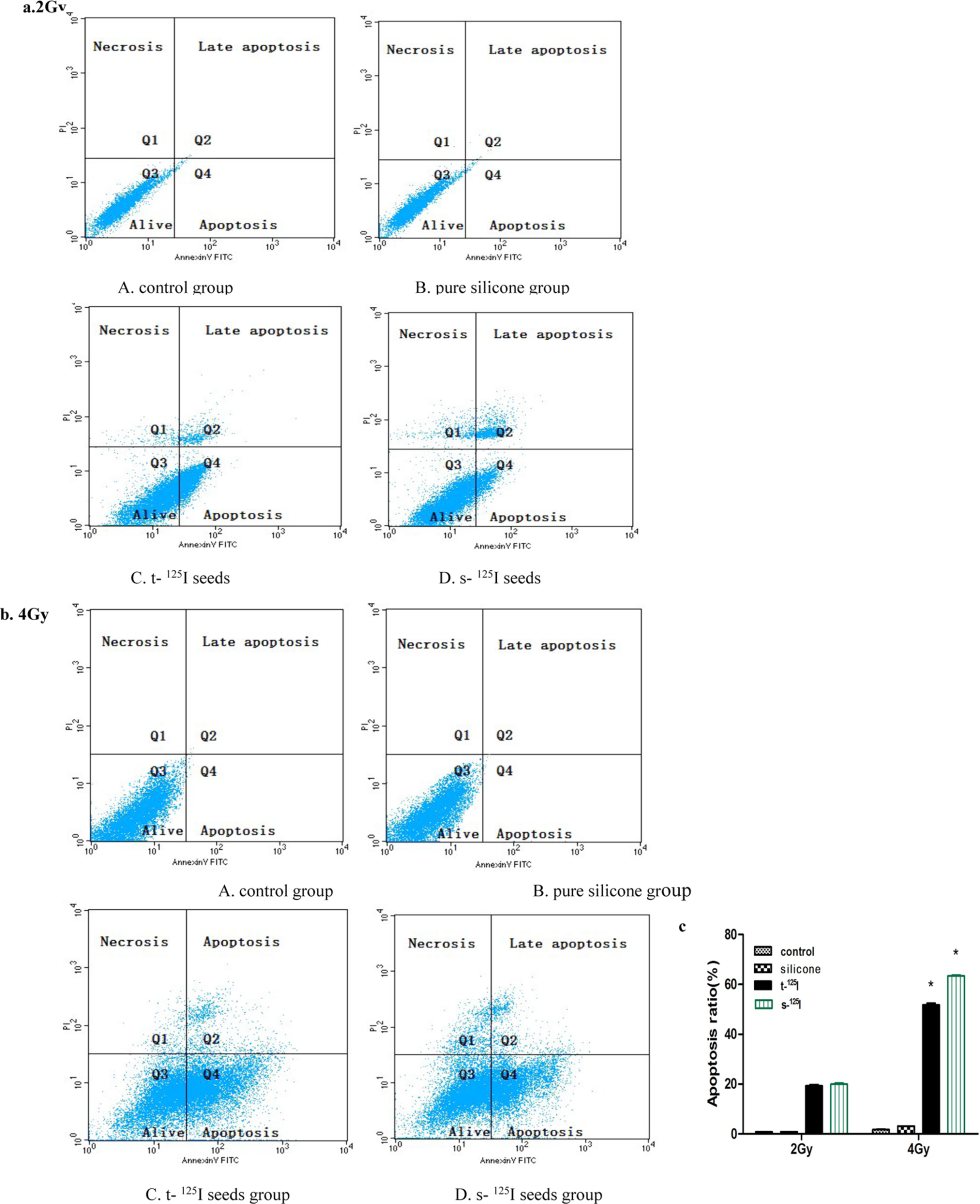
Evaluation of the in vitro anti-tumour effects of ^125^I seeds. (a) and (b)apoptotic progression in FRH-0201 cell lines in response to the titanium ^125^I seeds and silicone ^125^I seeds treatment for 2Gy and 4Gy; (c) cell FACS distributions (%) of apoptotic cells in different groups. Quadrant Q1,Q2,Q3 and Q4 reflect necrosis, late apoptosis, alive and early apoptosis, respectively.Total apoptosis includes late apoptosis plus early apoptosis. There was not significance between titanium ^125^I seeds and silicone ^125^I seeds groups. * P <0.05 means 4Gy compared with the 2 Gy groups respectively.

Tumor cells both in the control and silicone groups exhibited minimal apoptosis, while the ^125^I seed groups displayed an obvious increase in apoptosis. The percentage of apoptotic cells in the 2 Gy groups were 19.40% ± 0.64% for silicone ^125^I and 20.00% ± 0.56% for titanium ^125^I seeds(P>0.05). When cells were treated with 4 Gy, the apoptosis increased to 61.78% ± 0.90% for silicone ^125^I and 63.37% ± 0.44% for titanium ^125^I(P>0.05). Importantly, at either radiation dose, the silicone and titanium coated ^125^I exhibited a similar induction of apoptosis in tumor cells (Fig 6).

### 3.3 The inhibition of tumor growth

**Fig 7:**
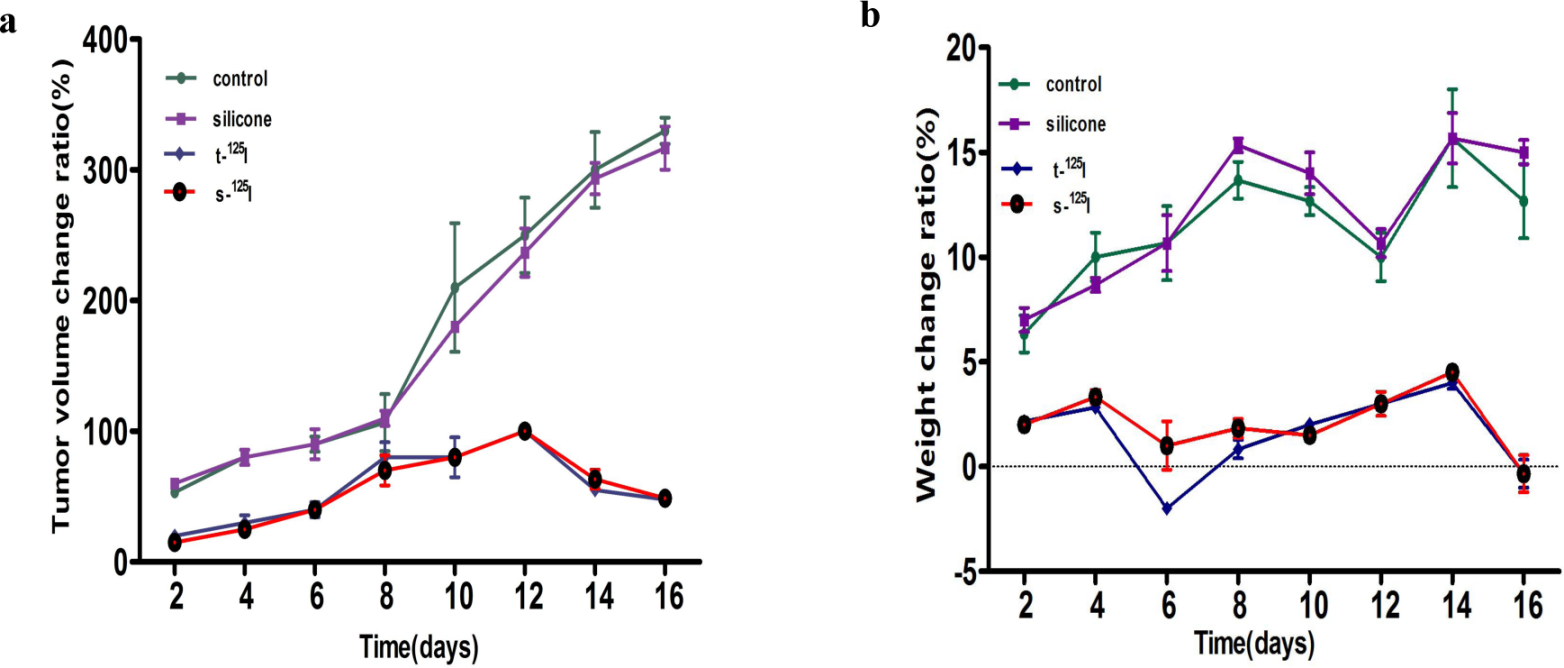
Investigation into the in vivo anti-tumor effects of the ^125^I seeds: (a,b)graphs of tumor growth and mice weight change after treatment with titanium ^125^I seeds and silicone ^125^I seeds in vivo.

No mice died during the experiment; however, one mouse in the titanium group lost the ^125^I seed. Our results showed that the tumor volume in the pure silicone group and control group increased rapidly over 2 weeks. Mice treated with either silicone or titanium coated ^125^I seeds show significant inhibition of tumor volume, with only a small increase in tumor volume (Fig 7A). Our observation of inhibition of tumor growth is consistent with the results of cell apoptosis by flow cytometry indicating that silicone and titanium ^125^I have similar therapeutic efficacy in vivo. The weight change ratio shown in Fig 7B illustrates that the silicone ^125^I seeds do not induce obvious side effects in vivo, because the mice weights remained stable.

### 3.4 Tissue staining analysis

**Fig 8:**
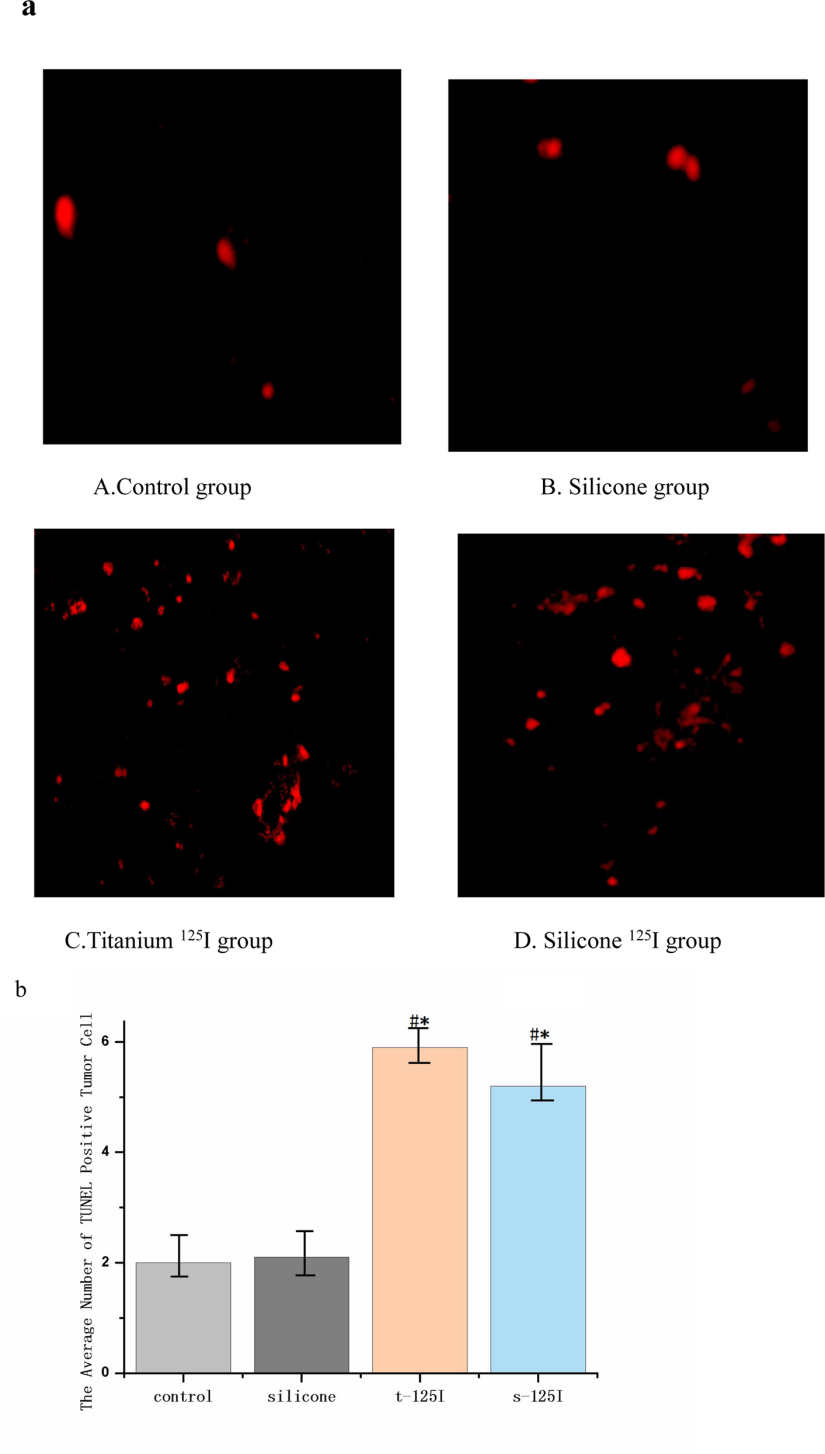
Anti-tumor effects of the ^125^I seeds by TUNEL staining:(a)the red spots represented the apoptotic cells detected by TUNEL staining in the each groups. The average number of apoptotic cells per 100 objective fields were plotted.(b) *P < 0.05 compared with the control group. #P < 0.05 compared with the pure silicone group.

Representative TUNEL stains obtained from the four groups are shown in Fig 8A. For either silicone or titanium ^125^I seeds, the average number of apoptotic cells was significantly higher than that for the control group (p < 0.05), but there was no statistically significant difference between the two coatings (Fig 8B). These data also suggest that the ^125^I seed implantation induced significant apoptosis in extrahepatic CC.

**Fig 9:**
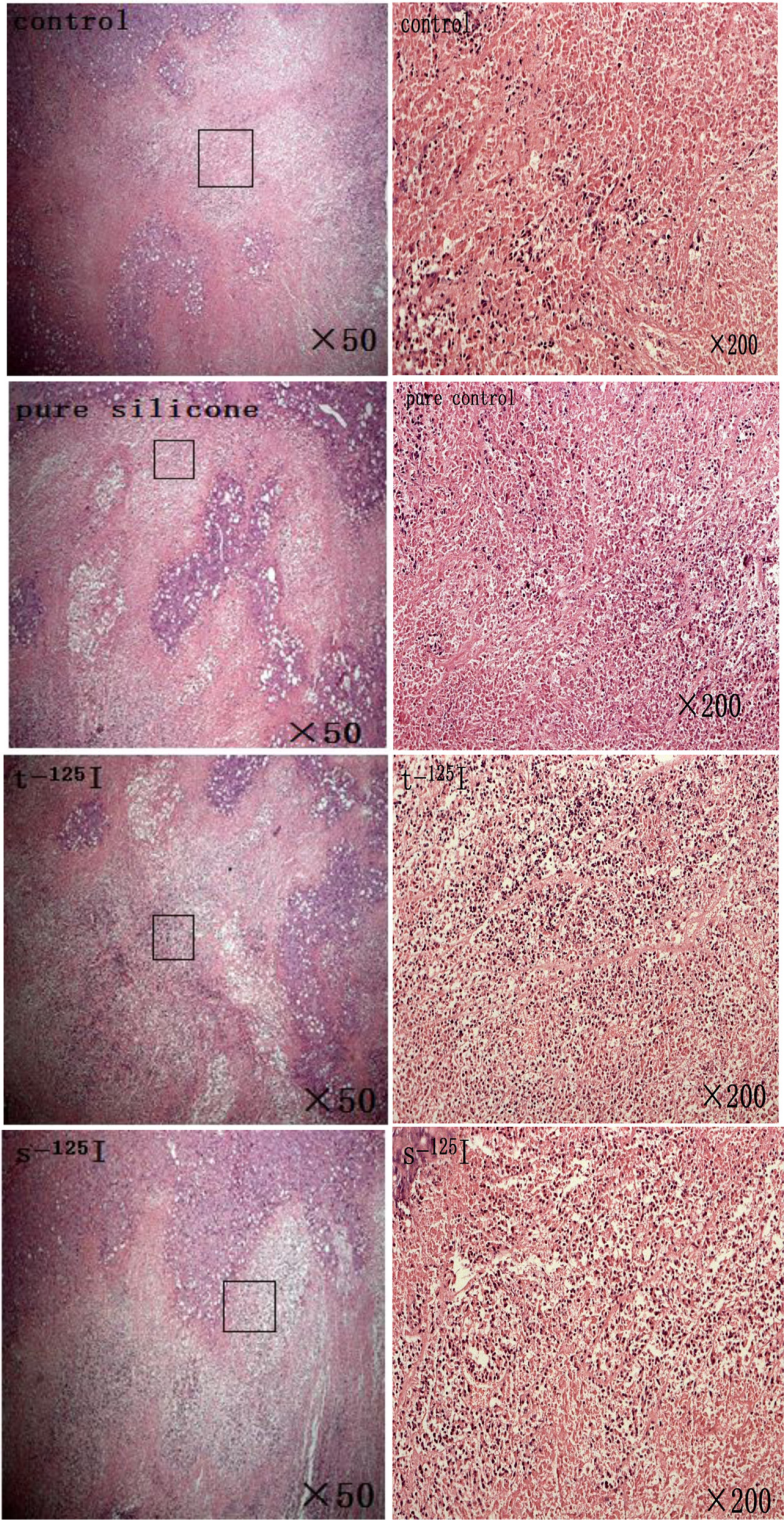
HE staining. The tumor histopathology for all ^125^I seeds and pure silicone groups after treatment. Tissue necrosis and ischemia were most frequently observed in the control and pure silicone groups, the silicone and titanium ^125^I groups showed very few necrotic or apoptotic cells were observed in the tumors.

Due to the rapid tumor growth in the control and pure silicone groups, tissue necrosis and ischemia were most frequently observed (Fig 9). However, the silicone and titanium ^125^I groups illustrated inhibition of tumor growth, and very few necrotic or apoptotic cells were observed in the tumors.

## Discussion

Biliary stent placement has been recommended as a palliative approach for the treatment of extrahepatic CC[2–7]. Several studies have demonstrated that stent insertion could relieve duct obstruction and maintain biliary drainage[22–26]. However, restenosis ignited by newly developed in-stent tumors is still an obstacle for this approach, and decreases survival[26–27]. To further control the growth of carcinoma, brachytherapy such as iridium 192 or holmium 166 in combination with stenting have been introduced. Although several studies showed favorable results, the efficacy of this approach remains controversial[11–14]. Furthermore, although holmium 166 and iridium 192 have been safely used for long-term brachytherapy, their half-life and penetration depth are short. In addition, an isolated, well-shielded room is needed for protection and to decrease the incidence of side effects to other organs when using iridium 192.

The ^125^I seed has a half-life of 59.6 days, and its whole failure period is about 400 days, can be implanted into the body, are under investigation as a promising brachytherapy source for carcinoma owing to their good treatment effect and low incidence of complications[14–16]. Several studies have demonstrated that implanting ^125^I seeds achieves satisfactory outcomes for unresectable pancreatic and hepatic carcinoma[28–29]; however, reports on extrahepatic CC are scarce because the bile tract anatomical structure makes implantation difficult to perform, and the titanium ^125^I seeds easily migrate. Thus, the radioactive seeds combined with biliary stenting may be an alternative approach. The ^125^I seeds coated by titanium are safety for radionuclide leakage and undegradable. However, the titanium-coated seeds are expensive, it is a burden for patients who need many seeds for the treatment in clinical, and due to the self-shielding effect, the radioactivity of ^125^I seeds weakens, and considerably more ^125^I solution is consumed during the procedure. Therefore, a long duration of treatment increases medical costs and requires good patient compliance. Medical silicone has been widely used for biomedical purposes. Compared to titanium, medical silicone also offers good tightness for ^125^I radionuclide, besides, including good biocompatibility, low cost and availability. Several studies achieved satisfactory outcomes by using silicone as biological drug delivery material[18–19].S. Radin et al. reported silica sol-gel showed biocompatibility and controlled resorbability of the drug composite in vivo, suggests that silica sol-gel is a promising carrier for the treatment of bone infections[30]. However, little research has been conducted into the use of silicone-coated ^125^I seeds for extrahepatic CC.

Although there is currently no adequate evidence to support the routine use of ^125^I radiotherapy postoperatively or for unresectable extrahepatic CC, important palliative value still have been reported. A epidemiological retrospective study in 4758 patients with extrahepatic CC suggested that palliative radiotherapy prolonged survival[31]. In our study, we developed a novel silicone-coated ^125^I seed. The silicone mixed with silver to form silver-silicone showed good absorption of ^125^I, and the silver-silicone is well encapsulated by silicone in combination with the technology of coaxial electrospinning. We found that the coating membrane was a nanofiber mixture, the ^125^I was stably distributed in the core, and the silicone ^125^I seeds have a higher utilization of ^125^I. Our study demonstrated that novel silicone ^125^I seeds inhibit extrahepatic CC with a similar efficacy as that achieved with titanium ^125^I seeds. In vitro experiments showed that the apoptosis rates were similar for the silicone ^125^I and titanium ^125^I seed groups. In vivo, control nude mice, which were not implanted with ^125^I seeds, showed rapid tumor growth after 16 days. In contrast, both the silicone ^125^I and titanium ^125^I seed groups exhibited significantly decreased tumor growth, and both groups showed similar tumor inhibition, Furthermore, the mice weights remained relatively stable throughout the experiment, because the ^125^I seeds effectively inhibited tumor growth. These datas demonstrate the feasibility of silicone ^125^I seeds as a palliative treatment for extrahepatic CC.

However, this study still has some limitations. First, although we measured the leakage of silicone ^125^I seeds to guarantee the safety of radionuclides in this procedure, it is an in vitro study, and the significance is limited by the nature of experimental research. Second, we established the xenografts on the back of mice as a model; however, the subcutaneous environment and the bile duct environment are different. The aim of our current study was to investigate the inhibition of tumor growth; however, our study lasted only for 16 days. As it is known, the significant role of silicone-coated ^125^I seeds are to be applied in the clinical work, treatment of patients, where the silicone ^125^I seeds are combined with a stent as a treatment for extrahepatic CC, may require much more time in vivo[24–25]. Therefore, a longer observation period is require, so far, we have successfully complete this novel silicone ^125^I seeds in combination with the placement of a stent in pig bile duct. The silicone ^125^I seeds performance, in vivo degradation, effects in the bile duct tissues of the model will be observed and analyzed in the future to ensure our model yields an accurate outcome.

## Conclusions

^125^I seeds coated with titanium are considered a safe and effective interstitial brachytherapy for tumors. In this work, ^125^I were mixed and coated with a modified coaxial electros-pinning machine showed silicone coating ^125^I seeds are straightforward to produce,. The results demonstrated that silicone ^125^I seeds showed higher radioactivity, compared to titanium ^125^I seeds. The results for the cell cycle and apoptosis evaluation showed that the silicone ^125^I seeds share the same anti-tumor effect with titanium ^125^I seeds, and the sustainable irradiation provided better anti-tumor effect. Furthermore, in vivo, the implanted titanium ^125^I seeds and the silicone ^125^I seeds demontrated the similar anti-tumor capability. Silicone-coated ^125^I seeds hold promise for treating extrahepatic CC. Further studies are needed to determine the efficacy and safety of silicone-coated ^125^I seeds in combination with biliary stenting in vivo.

## Supporting Information

S1 Fig Dates of mouse weight and volume change. (TIF)

S1 Table The table includes the minimal dates of cell apoptosis,the assay was performed 3 times for each experimental group. (XLS)
