# Supplementary figures and images for: Novel Silicone-Coated 125I Seeds for the Treatment of Extrahepatic Cholangiocarcinoma
Source: PLoS One. 2016 Feb 3;11(2):e0147701. doi: 10.1371/journal.pone.0147701 (PMC4740417; doi:10.1371/journal.pone.0147701)

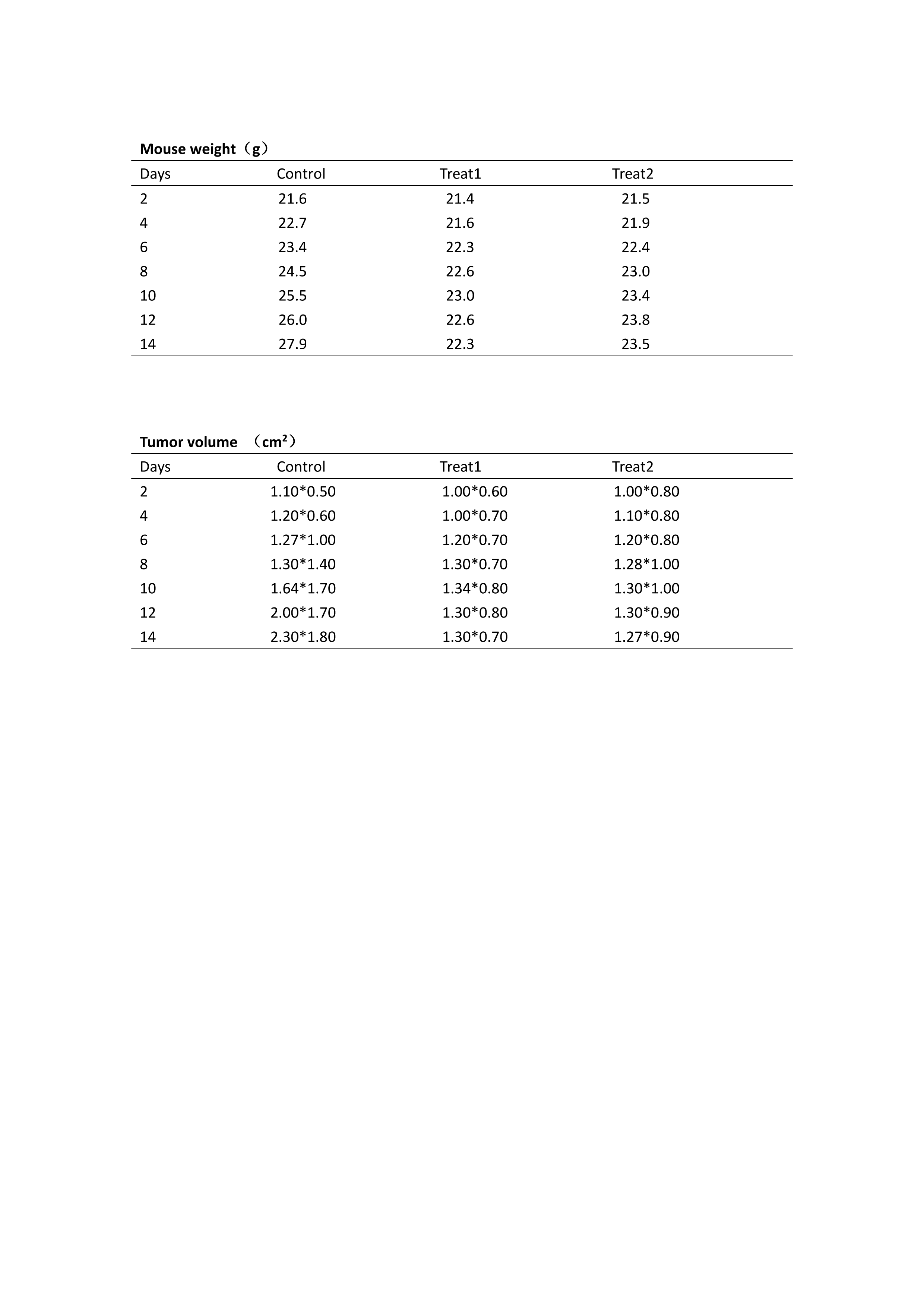

Supplement: S1 Fig — (TIF) [file pone.0147701.s001.tif]
